# Clinical and Diagnostic Features of 413 Patients Treated for Imported Strongyloidiasis at the Hospital for Tropical Diseases, London

**DOI:** 10.4269/ajtmh.19-0087

**Published:** 2019-06-17

**Authors:** Damien K. Ming, Margaret Armstrong, Patricia Lowe, Peter L. Chiodini, Justin F. Doherty, Christopher J. M. Whitty, Alastair C. McGregor

**Affiliations:** Hospital for Tropical Diseases, University College Hospitals London NHS Foundation Trust, London, United Kingdom

## Abstract

This study describes the clinical features of a cohort of imported cases of strongyloidiasis and the performance of standard diagnostic techniques for this condition. A total of 413 cases were identified, of whom 86 had microscopically proven infection. In proven cases, 23% had normal eosinophil counts, 19% had negative *Strongyloides*-specific serology, and 9.3% had normal blood counts and were seronegative. Serological testing was less sensitive for returning travelers (46.2%) than for migrants (89.7%). Immunosuppression, including human T-cell lymphotropic virus 1, was significantly associated with proven infection after controlling for age, presence of symptoms, duration of infection, and eosinophilia (OR 5.60, 95% CI 1.54–20.4). Patients with proven infection had lower serology values than those diagnosed with strongyloidiasis on the basis of positive serology and eosinophilia alone (*P* = 0.016). Symptomatic patients were significantly younger, had a shorter presumed duration of infection, and lower serology values. These data suggest a correlation between immunologic control of strongyloidiasis and the amplitude of the humoral response.

## INTRODUCTION

*Strongyloides stercoralis* is a parasitic nematode with a life cycle which includes both free-living and parasitic stages. *Strongyloides* can complete its entire life cycle in humans, and infection may, therefore, persist far beyond the life span of an individual helminth and endure until the death of the host.^1^ Infection is often asymptomatic.^2^ It is greatly in the interests of travelers to have their *Strongyloides* diagnosed early; it can cause unpleasant symptoms, and occasionally massive and uncontrolled multiplication of *Strongyloides* parasites, which usually occurs in the context of immunosuppression, can lead to a severe hyperinfection syndrome with a mortality rate approaching 70%.^3^

The diagnosis of strongyloidiasis is complicated as symptoms of infection, when present, are often nonspecific. Traditional microscopic and culture techniques are known to lack sensitivity because of low worm burden and intermittent shedding in stool.^4,5^ Molecular assays based on nucleic acid detection hold promise^6^ but are currently not in routine clinical use and may be more suitable as confirmatory rather than screening tests.^7^ Serological techniques using *Strongyloides* spp. antigen have a higher sensitivity than stool examination,^8^ although specificity may differ significantly in different patient groups.^9^ Furthermore, serological tests cannot reliably distinguish active from past infection.^10^ In the absence of a gold standard test, the performance characteristics of serological assays for active disease or the role of serological assays in treatment follow-up remains poorly defined. Some clinics use eosinophilia as a screening tool.

The relationship between the clinical presentation and diagnostics, including serology and culture, in strongyloidiasis has not been systematically examined in a large cohort. We performed an audit of 413 consecutive outpatients treated for *Strongyloides* at the Hospital for Tropical Diseases (HTD), London, between 1999 and 2017. This group of travelers and migrants, in whom infection was presumed to have occurred overseas, offers a unique opportunity to assess the features of strongyloidiasis in a non-endemic setting with the aim of identifying factors that clinicians confronted with patients who returned from the tropics should consider in their diagnostic approach.

## METHODS

Cases were identified by retrospective analysis of prospectively coded data, and clinical information was obtained by review of pseudo-anonymized clinical and laboratory data at the HTD. A case of strongyloidiasis was defined as proven (confirmed through microscopy and/or culture) or presumed (positive serological test, without previous treatment). *Strongyloides* serological testing was performed using a commercial ELISA based on antigen derived from *Strongyloides ratti* (Bordier, Switzerland), with results expressed as the ratio of optical density (OD) value/cutoff. A positive serology result was defined as a value greater than 1. Patients were classified as travelers if they were born and predominantly reside in a non-endemic country for strongyloidiasis, and were classified as migrants if they were born in or predominantly reside in a *Strongyloides*-endemic country. Patients were considered immunocompromised if they were receiving immunosuppressive medications (cancer chemotherapy, transplant rejection medication, and steroids) or they had an identifiable immunocompromising medical condition such as HIV or human T-cell lymphotropic virus 1 (HTLV-1) infection. Clinical symptoms were recorded according to the system affected and for the purposes of analysis were grouped together as present or absent. Stool and charcoal culture was performed using techniques previously described.^11^ Eosinophilia was defined as a peripheral blood absolute eosinophil count of ≥ 0.5 × 10^9^/L. Statistical analyses were performed on anonymized data using Stata v.14 (StataCorp, College Station, TX) with chi-squared and Mann–Whitney testing, as appropriate. Logistic regression was performed with missing data substituted with dummy variables.

## RESULTS

The clinical characteristics and diagnostic results of 413 patients treated are summarized in Table 1.

**Table 1:** Clinical and laboratory characteristics of patient cohort treated for strongyloidiasis (*n* = 413)

Characteristic	number	%
Median age in years (interquartile range)	48 years (range 36–61 years)	
Female (*n*)	158	(38)
Migrant/traveler status known (*n* = 400)		
Patients classified as migrants	315/400	(78.8)
Patients classified as travelers	85/400	(21.3)
Primary presenting symptoms (*n* = 405)		
Gastrointestinal	131	(32.3)
Dermatological	32	(7.9)
Respiratory	15	(3.7)
Other	38	(9.4)
Asymptomatic	189	(46.7)
Underlying immunosuppression (*n* = 48)		
Immunosuppressive medications including chemotherapy	8	(16.7)
Steroid use	8	(16.7)
HIV infection	12	(25)
HTLV-1 infection	7	(14.6)
Other	13	(27.1)
Positive *Strongyloides* microscopy/culture (*n* = 86)		
Positive on stool microscopy alone	42/86	(48.8)
Positive on charcoal culture alone	29/86	(33.7)
Positive on both stool microscopy and charcoal culture	15/86	(17.4)
Overall sensitivity of eosinophilia in cases confirmed by microscopy/culture	59/77	(76.6)
Sensitivity of eosinophilia in migrants	46/60	(76.7)
Sensitivity of eosinophilia in travelers	10/13	(76.9)
Overall sensitivity of serology in cases confirmed by microscopy/culture	70/86	(81)
Sensitivity of serology in migrants	61/68	(89.7)
Sensitivity of serology in travelers	6/13	(42.2)

### Demography, travel history, and clinical features.

**Figure 1.**
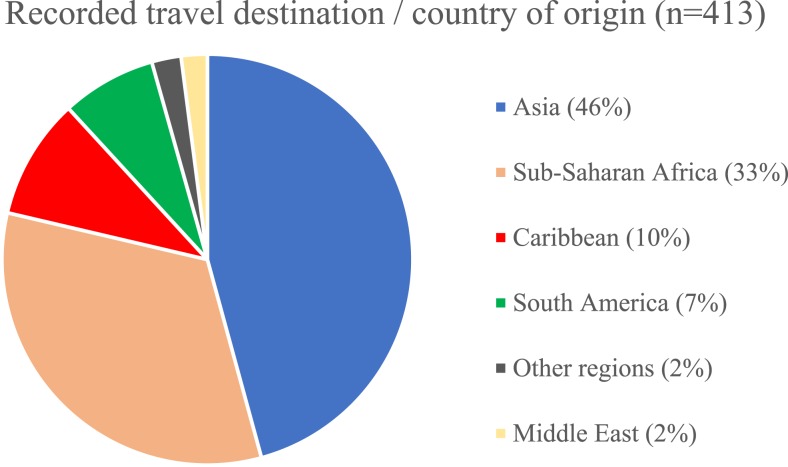
Travel destination and country of origin of patients between 1999 and 2017. This figure appears in color at www.ajtmh.org.

The travel destinations and areas of origin of the patients were diverse, but Asia (45.8%) and sub-Saharan Africa (32.9%) were the most commonly featured regions (Figure 1). When the region of origin was stratified into 6-year periods, migrants originating from Asia increased overall with time (43.7% to 51.5%), whereas those from sub-Saharan Africa decreased (39.0% to 25.8%). The median age of patients increased over time (range 41–49 years). Most patients presented with gastrointestinal symptoms (32%), which included abdominal pain, vomiting, and a change in bowel habit. Around 6.3% of patients presented with larva currens as their primary symptom, and 1.6% of patients with rash and itching. A small proportion (3.7%) of patients presented with respiratory symptoms,which include cough, shortness of breath, and chest discomfort. Almost half (47%) of the patients were asymptomatic. Patients with symptoms were younger (median age 43 versus 54 years, *P* < 0.001). Patients classified as travelers were also more likely to be symptomatic than migrants (74% versus 48%, *P* < 0.001). In a logistic regression model after controlling for age, serology result, and the presence of eosinophilia, only duration of infection in years remained significantly associated with the presence of symptoms, and the association was small (OR 0.97, 95% CI 0.96–0.99, *P* = 0.01). Within the cohort, 38% (159/413) patients also underwent serological testing for schistosomiasis, but positive results were only seen in 6.9% (11/148).

### Performance of microscopy and serology.

Eighty-six patients (21% of the cohort) had a proven diagnosis of strongyloidiasis based on positive stool microscopy or culture. The majority (76.7%) of the proven cases were associated with a peripheral blood eosinophilia, and just more than half (54.7%) of this group were symptomatic. *Strongyloides* serology was positive in 70/86 patients, giving an overall test sensitivity of 81% in this cohort. Forty-two (48.8%) of the proven cases were diagnosed on stool microscopy alone, 29 cases (33.7%) on charcoal culture alone, and 15 cases (17.4%) had a positive stool microscopy and charcoal culture. Of particular note, eight (9.3%) patients with proven infections had neither a positive serology nor eosinophilia.

### Migrants versus travelers.

Within the parasitologically proven group, individuals classified as migrants were much more likely to be seropositive (89.7%, 61/68 positive) than those classified as travelers (46.2%, 6/13 positive, *P* < 0.001). The median (IQR) serology value was higher for migrants than travelers in this group: 1.99 (range 1.34–2.80) versus 0 (range 0–1.72), respectively (*P* = 0.022). Migrants were more likely to have a positive serology (OR 10.2, 95% CI 2.67–39.0, *P* = 0.001) when age, gender, presence of symptoms, and immunosuppression were accounted for in a logistic regression model.

### Magnitude of serological response.

The median serology (IQR) value in patients with proven *Strongyloides* infections was significantly lower than that in patients with a presumed diagnosis (based on positive serology and eosinophilia, but negative microscopy/culture): 1.87 (1.10–2.71) versus 2.25 (1.52–3.25), respectively (*P* = 0.016). The presence of symptoms was also associated with a lower serology value at diagnosis compared with asymptomatic patients (median OD/cutoff: 1.56 versus 1.94, *P* = 0.003).

### Immunosuppression.

A higher proportion of patients with proven infection had an underlying immunosuppressive condition compared with patients with a positive serology alone (23.0% versus 9.7%, respectively, *P* < 0.001). Immunosuppression remained significantly associated with positive *Strongyloides* microscopy/culture in multiple logistic regression analyses after age, duration of infection, presence of symptoms, and eosinophilia were taken into account (OR 5.60, 95% CI 1.54–20.4, *P* = 0.009). None of the five patients who tested positive for HTLV-1 with proven *Strongyloides* infections presented with peripheral blood eosinophilia at diagnosis. The median serology value for patients with HTLV-1 infection was also lower than that for patients negative for HTLV-1 infection, although a significant difference was not demonstrated (0.75 versus 2.4, respectively, *P* = 0.32).

### Treatment.

Most patients (96.6%) were treated with ivermectin. Follow-up data were available for 280 (68%) patients, and the median follow-up time was 119 days after treatment. Of the patients who presented with eosinophilia, 24% (34/140) patients had persistent eosinophilia after treatment. Nearly half of patients treated (80/167) had negative serology at follow-up. Overall, the great majority (84%, 124/148) of patients had a lower *Strongyloides* serology value after treatment on follow-up, and about two-thirds (63%) had a decrease in ELISA OD of greater than half.

## DISCUSSION

Diagnosing *Strongyloides* early is in the interests of travelers, but screening and diagnostic strategies have to take account of the limitations of diagnostic methods. We describe the important clinical and laboratory features of a large cohort of individuals with strongyloidiasis who were treated at the HTD, London, over a period of 18 years. In patients with infection proven through stool microscopy or culture, nearly a quarter (23%) did not have peripheral blood eosinophilia and more than half (54.7%) were asymptomatic, implying neither symptoms nor eosinophilia is sufficiently sensitive for screening. Nonetheless, eosinophilia remains an important diagnostic feature and its presence warrants further investigation for parasitic infections. In our group, a quarter of patients who were followed up had persistent eosinophilia after treatment for strongyloidiasis. In these cases, further evaluation for other causes including parasitic coinfections is carried out, and testing was performed according to the geographical region of travel or origin.^12^

The seropositivity rate in this group of microscopically proven infections, at 81%, was lower than published sensitivity data^13^ and was significantly lower still in travelers (46.2% compared with 89.7% in migrants), observations consistent with previous findings in our hospital.^9^ In a significant number (9.3%) of proven infections, strongyloidiasis was not suspected at all because of both negative serology and the absence of a peripheral blood eosinophilia (in these cases, stool testing was performed to look for protozoan gastrointestinal pathogens). There is a possibility that serology would be more specific with recombinant antigens than crude antigens, although this is unlikely fully to explain the difference between travelers and migrants, given most have been long-standing.

Given the limitations of both serological and microscopic tests for *Strongyloides* infection, a low diagnostic threshold should be maintained. Intermittent shedding of parasites in stool is long recognized, and repeat stool tests are probably more sensitive than single tests.^14^ Charcoal culture is also useful in our cohort; this technique more than doubled the diagnostic yield compared with the use of fecal concentration and microscopy alone. Although charcoal culture is not widely used, it is inexpensive to perform,^11^ and we recommend its use when strongyloidiasis is suspected. Other simple methods for improving diagnostic yield from stool include the use of agar plate culture, which has shown to improve sensitivity over methods such as conventional direct examination.^15^ Newer techniques, such as ELISA-based *Strongyloides* coproantigen detection^16^ and various PCR-based molecular tests,^17–21^ remain experimental but show promise as sensitive tests. However, PCR-based assays may be more suitable as confirmatory tests than initial screening^7^ for this pathogen.

Our study is limited by its retrospective nature and the fact it is from a single tertiary center. Patients were referred as a result of symptoms related to travel or unexplained eosinophilia and, therefore, may not be representative of a normal population. The distinction between travelers and migrants was based on country of birth, which is somewhat simplistic, but any resultant inaccuracies would tend to reduce the differences between travelers and migrants rather than create them. The duration of infection, especially in the traveler group, was inferred by the date of most recent travel. We had incomplete follow-up data and acknowledge that limited conclusions can be drawn from these results, which are at risk of selection bias; we have, therefore, concentrated on presentation data. Follow-up data, such as including residual symptoms and eosinophilia, were also incomplete and may overrepresent patients who had persistent symptoms and/or another diagnosis.

Most importantly, as with all *Strongyloides* research, our ability confidently to diagnose active infection was hampered by the lack of a gold standard test. We were only able to assume probable infection in a significant number of patients, and this limits our ability to accurately estimate the sensitivity of serology, a problem for most studies of *Strongyloides* as there is no widely accepted gold standard. More patients underwent serological testing (93%) compared with stool microscopy/culture (68%). This discrepancy is due to the outpatient nature of the clinic in which patients could undergo serological testing immediately but often had to submit stool specimens at a later date, leading to a drop-off in numbers. On the other hand, our cohort is one of the largest described in a non-endemic setting, and the virtue of this single-center study is that diagnostic strategies (while limited) and treatment algorithms were relatively standardized and reflect clinical reality in centers in high-income settings.

A robust immunity including a Th2 response is an essential component in the control of infection.^22,23^ Our data show an inverse relationship between the likelihood of microscopic proof of infection (as a proxy of high-burden infections) and the strength of serological response (as measured by the ELISA result). We speculate that newer infections may be associated with a high parasite burden, reflected by the higher chance of a positive parasitological test, and increased probability of symptoms. With immune control over time associated with increasing serological levels, the opposite is true.

In conclusion, we demonstrate important clinical and laboratory factors in strongyloidiasis within a real-life cohort of patients over a period of 18 years in a non-endemic area. We found that the performance of standard diagnostic tests such as stool analysis, serology, and blood count varied according to the presumed duration of infection, the presence of symptoms, and the presence of immunosuppression, and in particular, serology and blood eosinophilia were both of limited sensitivity in travelers. Improved techniques for confirming active infection—a gold standard worthy of the name—are required for accurate diagnosis and optimal management of this infection.
